# Sugar slay: a gamified decision support ecosystem for type 1 diabetes

**DOI:** 10.3389/fdgth.2026.1779790

**Published:** 2026-06-17

**Authors:** Sundararaman Rengarajan, Nicholas Abrams, Aspen Tabar, Hariharan Sundaram, Kavya Pratap Singh, Leanne Chukoskie

**Affiliations:** 1 Bouvé College of Health Sciences, Northeastern University, Boston, MA, United States; 2 Khoury College of Computer Sciences, Northeastern University, Boston, MA, United States; 3 College of Engineering, Northeastern University, Boston, MA, United States; 4 College of Professional Studies, Northeastern University, Boston, MA, United States; 5 College of Arts, Media and Design, Northeastern University, Boston, MA, United States

**Keywords:** Type 1 Diabetes, digital Health, decision support system, glucose prediction, gamification, habit formation, mobile health, machine learning

## Abstract

**Background/Introduction:**

Type 1 Diabetes (T1D) management demands consistent attention to blood glucose levels, insulin dosing, physical activity, sleep, and diet-tasks that are especially burdensome for adolescents and young adults navigating new independence. While continuous glucose monitoring (CGM) systems and wearable fitness devices provide real-time physiological data, the cognitive load of interpreting this information and maintaining consistent self-care behaviors remains a significant barrier for patients and their support networks.

**Methods:**

We developed the Sugar Slay ecosystem, comprising a gamified mobile decision support application for T1D individuals and a companion application, *Sugar Slay Care*, for caregivers and supporters. The main application integrates CGM and wearable device data to generate real-time, personalized predictions using advanced machine learning models, including a Sequence-to-Sequence Bidirectional LSTM (Seq2Seq BiLSTM). To inform the design of *Sugar Slay Care*, we conducted a need-finding study with six caregivers and supporters to identify features critical to balancing autonomy with safety.

**Results:**

Among the machine learning models evaluated, the Seq2Seq BiLSTM demonstrated the best performance in forecasting blood glucose trends. The need-finding study identified key caregiver requirements, informing the development of a companion application that supports safety monitoring without undermining patient independence.

**Discussion:**

Sugar Slay integrates predictive modeling with habit-building gamification strategies to encourage daily engagement and proactive self-management. By addressing the social dimension of T1D care through *Sugar Slay Care*, this work contributes to next-generation digital health tools that unite physiological data, artificial intelligence, and behavioral science for user-centered chronic disease management.

## 1 Introduction

Managing Type 1 Diabetes (T1D) requires consistent monitoring and control of blood glucose levels to reduce the risk of complications. Advances in Continuous Glucose Monitoring (CGM) systems, insulin pumps, and wearable devices for physiological monitoring have significantly improved diabetes management (1–5). These tools provide real-time insights into important health parameters such as glucose levels (6, 7), Heart Rate Variability (HRV) (8), sleep scores (9), recovery rates (10), and calories burned (11). However, despite the availability of these advanced technologies, challenges remain in integrating and interpreting the vast amounts of data produced to optimize glycemic control and personalize treatment strategies.

Furthermore, T1D management does not occur in a vacuum; it deeply impacts the social ecosystem surrounding the patient. Supporters—parents, siblings, and friends—often struggle to balance their desire to help with the patient’s need for autonomy. This paper presents the development of the **Sugar Slay Ecosystem**, a comprehensive solution comprising two interconnected applications: the main *Sugar Slay* app for T1D individuals and the *Sugar Slay Care* companion app for their supporters.

We present three complementary studies that inform this ecosystem:

1. A machine learning model evaluation study using the OhioT1DM dataset to determine the best performing predictive algorithm for glucose forecasting.
2. An iterative user experience study with 7 T1D participants to assess the main app’s usability and gamification features.
3. A qualitative need-finding study with 6 caregivers and supporters (parents, siblings, and friends) to determine the specific functional requirements for the companion app.

While CGM and insulin pump systems offer real-time data, the interpretation and application of this information is often left to users, which can be overwhelming and lead to sub-optimal management decisions (12). This highlights the need for advanced decision support tools (DSTs) that can intelligently integrate and analyze data from multiple sources.

Emerging research supports the potential of AI-driven DSTs and event-triggered systems for improved glycemic control (13–15). Adolescents with T1D, in particular, face unique challenges such as developmental behaviors and family dynamics, which negatively impact adherence (16). Through our machine learning study, we demonstrate the potential of various algorithms to predict glucose trends. Our usability testing reveals strong user acceptance of gamification. Finally, our interviews with supporters reveal a critical need for “peace of mind” tools that respect patient privacy—a gap we address with *Sugar Slay Care*.

These initial findings represent an important step toward the development of a unified tool that simplifies the management of T1D for the patient while intelligently integrating their support system.

## 2 Background

The management of T1D has been greatly enhanced by the development of CGM systems, insulin pumps, and smart insulin pens, which help automate aspects of glucose monitoring and insulin delivery (2, 3, 5). However, even with these advances, the interpretation of data and decision-making process can still result in inefficiencies and errors, not solely due to user involvement but also because current algorithms do not yet fully account for contextual factors (14, 15). This limitation can contribute to glucose variability (GV) oscillations, as both the user and the system struggle to maintain optimal glucose levels (12). In addition, the integration of other personal health metrics such as physical activity (11), HRV (8), sleep quality (9, 17), and dietary intake (18, 19) provides a more holistic perspective on a person’s health, which is crucial for effectively managing diabetes.

Heart Rate Variability (HRV) is an important indicator of autonomic function and stress resilience. In T1D management, HRV can reflect the body’s response to fluctuating glucose levels, with lower HRV signaling potential stress or health issues (20). Research demonstrates that HRV parameters are significantly reduced in T1D patients, serving as sensitive early markers for cardiac autonomic neuropathy (21, 22). Poor glycemic control can lead to autonomic neuropathy, which reduces HRV, making it a valuable metric for identifying periods of physiological stress that may precede glucose variability (20). Remarkably, HRV changes enable hypoglycemia prediction, with algorithms combining CGM and HRV achieving high sensitivity and specificity (23, 24).

Sleep is another critical factor affecting glucose metabolism. Insufficient sleep can result in insulin resistance and higher glucose variability, complicating blood sugar management (17). Studies show that even a single night of partial sleep restriction reduces insulin sensitivity by 21% in T1D patients (17). Sleep deprivation also increases insulin demand and worsens hormonal imbalances, making it harder to maintain stable glucose levels (25). Recent research reveals complex bidirectional relationships between sleep and glucose control, with each 1% decrease in sleep efficiency associated with reduced time in range (25). Tracking sleep patterns allows individuals to better understand these dynamics and adjust their diabetes management strategies accordingly (9, 26, 27).

Physical activity plays a vital role in regulating glucose levels by increasing insulin sensitivity, which allows the body to use glucose more efficiently. However, the relationship between physical activity and glucose levels is complex, with different types of exercise (aerobic vs. anaerobic) having varying effects on blood sugar (11). The Type 1 Diabetes Exercise Initiative demonstrates that aerobic exercise causes the largest glucose drops, while resistance exercise produces smaller decreases with lower hypoglycemia risk (28). High-intensity interval training offers unique advantages through increased counter-regulatory hormone release (29). By analyzing exercise data, users can better predict how their physical activities influence their glucose levels and make more informed decisions about insulin dosing and carbohydrate intake (11, 30).

Diet is, of course, central to T1D management, as the timing, type, and quantity of food consumed directly affect glucose levels. Carbohydrates, in particular, have the most significant impact, but understanding the glycemic index and how different macronutrients interact can help manage blood sugar (18). Research shows that fat and protein significantly modify postprandial glycemia, with effects lasting up to 12 h post-meal (31). Meta-analyses confirm that carbohydrate counting reduces HbA1c by 0.49% (19), while low-glycemic index diets effectively reduce HbA1c, fasting glucose, and BMI (32). For example, high-fiber foods may stabilize glucose levels, while sugary or high-carb foods can cause spikes that require precise insulin management (33).

The integration of diverse data sources through machine learning presents a promising opportunity to enhance DSTs. While research has established general trends about how sleep patterns (17, 25), exercise routines (11, 28), and food consumption (18, 31) impact glycemic control, there is considerable individual variability in these relationships. What causes a glucose spike in one person may have minimal effect on another, and the same exercise intensity can produce dramatically different glycemic responses across individuals (28, 29). Similarly, sleep disruption affects insulin sensitivity differently across individuals (26), and macronutrient effects on postprandial glycemia can vary significantly from person to person (31). This heterogeneity makes discovering one’s personal patterns and proclivities particularly powerful.

By analyzing large datasets at the individual level, machine learning models can identify these person-specific patterns and predict trends, offering truly personalized diabetes management advice rather than generic recommendations (13–15). This individualized approach is what makes apps like Sugar Slay especially valuable—they enable users to understand their unique physiological responses and reduce the cognitive burden of constantly interpreting their own data, making the decision-making process more efficient and effective (34). Sugar Slay builds on these principles by incorporating predictive models that analyze historical and current data from CGM systems and other physiological sensors to provide preemptive management suggestions. Our preliminary results from both the machine learning evaluation and user experience testing demonstrate the potential of these models in stabilizing glucose levels and reducing the likelihood of acute glycemic events while maintaining high user engagement. The app’s gamification elements, validated through our usability study, show promise in improving engagement and adherence (35–38). Although we are at an early stage of development and a full pilot study is yet to be conducted, these initial findings underscore the promise of machine learning combined with user-centered design in supporting more personalized, data-driven diabetes management. Future iterations of the app will include formal testing and expanded user studies to validate its effectiveness in real-world settings (39).

## 3 Machine learning models for decision support in type 1 diabetes management

Machine learning (ML) models are pivotal in enhancing decision support tools (DST) for Type 1 Diabetes (T1D) management. These models integrate data from various sources, such as glucose levels from continuous glucose monitors (CGM), insulin dosages, and physiological parameters to provide personalized management recommendations (40). One of our goals in this paper is to compare different models to predict glucose levels in adolescent T1D data, aiming to use these models as a foundation for personalization with individual CGM and wearable physiological sensor data.

### 3.1 Model-based and data-driven algorithms

Physiologic and data-driven models, such as Model Predictive Control (MPC) and data-driven techniques like ARX (autoregressive models with exogenous inputs) and SVM (support vector machines), predict glucose levels and insulin responses (41–43). These models are essential for creating initial predictions that can be fine-tuned using specific adolescent data, allowing DSTs to evolve based on real-time personal sensor readings.

### 3.2 Advanced learning methods

Reinforcement and adaptive learning strategies continuously optimize insulin dosing by updating algorithms with new data. Clustering methods like K-nearest neighbors (KNN) and Case-Based Reasoning (CBR) use historical and current patient data to enhance decision-making (44). These techniques are vital for adapting base models to reflect the unique physiological characteristics of adolescents.

### 3.3 Hybrid and rule-based systems

Hybrid models integrate various computational approaches to leverage their strengths, enhancing predictions with deeper insights from machine learning. Rule-based systems, including fuzzy logic, interpret sensor data to make personalized insulin recommendations. These systems adjust their rules based on real-time data, providing highly personalized treatment options for adolescents (45, 46).

These diverse ML models significantly contribute to the advancement of DST for T1D, aiming to improve life quality by reducing management burdens and enhancing glycemic control. The integration of adolescent-specific data and the capability for model personalization using real-time user data underscore significant progress in applying machine learning to manage diabetes effectively.

## 4 Data collection and pre-processing

Our study employed a two-phase approach to develop and implement the Sugar Slay system. In the first phase, we utilized the OhioT1DM dataset (47) exclusively for machine learning model development and evaluation. This modeling effort served as a preliminary study to identify the most effective predictive algorithms for glucose forecasting, helping us determine the best performing model architecture for real-time implementation. The insights gained from this analysis informed our selection of the most suitable machine learning approach for the Sugar Slay application.

In the second phase, the Sugar Slay application integrates two primary real-time data streams: the WHOOP fitness tracker for continuous physiological monitoring and the Dexcom G6 continuous glucose monitor for real-time glucose tracking. These two data sources form the foundation of the app’s operational framework, providing the continuous health metrics necessary for personalized glucose predictions and recommendations. Unlike the OhioT1DM dataset, which was used solely for model selection and validation, the WHOOP and Dexcom data streams are actively processed by the app to deliver real-time insights to users. This multi-source integration demonstrates our system’s ability to synthesize diverse health data streams while maintaining data integrity and security.

### 4.1 OhioT1DM dataset for model development

For the machine learning model evaluation study, we utilized the OhioT1DM dataset (47), which consists of comprehensive T1D management data for 18 subjects, with 12 used for training and 6 for testing. This dataset was originally recorded in XML format and converted to CSV to maintain the precision of the time-series data, crucial for the analysis. Each record in the dataset includes time-stamped entries of glucose levels, insulin dosages (both basal and bolus), and other physiological markers that are essential for diabetes management. The OhioT1DM dataset was used as a standardized benchmarking dataset for model comparison because it is widely adopted in glucose forecasting research and enables reproducible evaluation against prior literature. The current study represents an early-stage model development and validation phase focused on architecture comparison rather than deployment-specific optimization.

### 4.2 Real-time data integration from wearable devices

To demonstrate the app’s real-world data integration capabilities, we implemented connections to both the WHOOP fitness tracker and Dexcom G6 CGM system. The WHOOP device, through its API, enables the seamless collection of continuous physiological measurements such as heart rate variability, recovery metrics, sleep quality, and physical activity data. Similarly, the Dexcom G6 provides real-time glucose readings at 5 min intervals, along with trend information and rate of change data. By leveraging these APIs, our React Native application integrates these complementary metrics, facilitating real-time updates and data storage on Cloud Firestore (HIPAA compliant). This integration ensures data security through OAuth authentication for both devices, allowing for a robust and secure user experience that combines glucose data with physiological context.

### 4.3 Data pre-processing

In the pre-processing phase for the OhioT1DM dataset, the data concerning “basal” and “temp basal” insulin rates were merged into a single parameter to simplify the analysis. Here, the “basal” refers to the continuous rate of insulin infusion that begins at a specified timestamp and remains effective until a new rate is specified. The “temp basal” is a temporary rate that overrides the normal basal rate; a value of 0 in this parameter signifies a suspension of basal insulin delivery. Once the temporary rate period concludes, the insulin rate automatically reverts to the regular basal rate. This method of consolidation allows for more streamlined data analysis. Data conversion efforts were aimed at preserving the original time intervals without employing interpolation methods, ensuring the integrity of the temporal data. Missing values in the dataset were addressed by filling non-glucose columns with zeros, whereas glucose values were left untouched to avoid introducing inaccuracies in the predictive models. While the WHOOP and Dexcom G6 data demonstrate our system’s integration capabilities, the machine learning models presented in this paper were trained and evaluated exclusively on the OhioT1DM dataset to ensure reproducibility and scientific rigor. Future work will involve personalizing these models using individual user data from WHOOP and Dexcom devices.

## 5 Technology integration and user workflow in the sugar slay application

### 5.1 Technology stack

To support the gamified decision support tool, Sugar Slay leverages a robust technology stack designed to ensure a seamless and interactive user experience. The application is built using React Native, allowing for cross-platform compatibility across iOS and Android devices. Connectivity with the WHOOP Health Tracker and Continuous Glucose Monitoring (CGM) systems is facilitated through Bluetooth, enabling real-time data transmission directly to the user’s mobile device.

**Figure 1:**
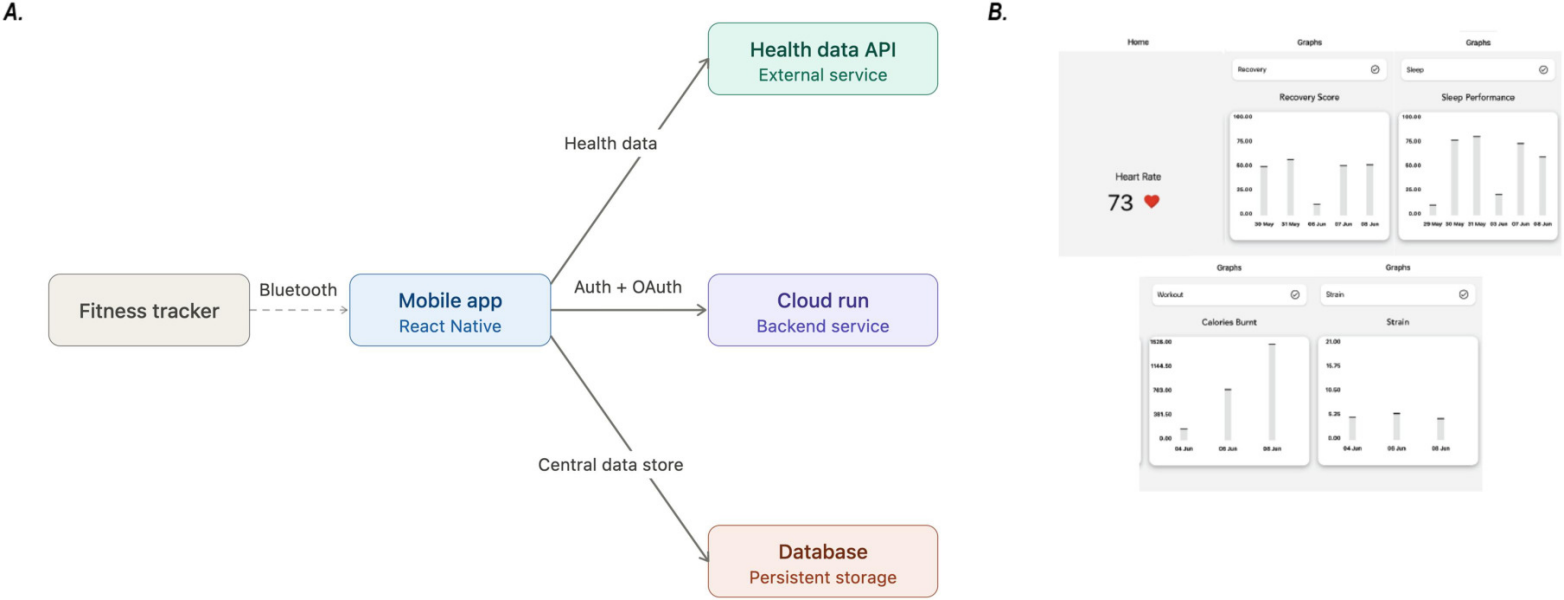
Integration of wearable technology and application interface. **(A)** Schematic representation of the data flow architecture connecting the WHOOP fitness tracker to the Sugar Slay React Native mobile application. The system captures live heart rate data via Bluetooth and synchronizes broader health metrics through the WHOOP API, utilizing Google Cloud Run for authentication and Firestore for centralized storage. **(B)** Application screenshots demonstrating the visualization of physiological data, including real-time heart rate monitoring alongside longitudinal graphs for recovery, sleep performance, caloric expenditure, and physiological strain.

For backend services, Google Cloud Run is utilized for deploying server-side components, ensuring scalable and efficient management of application processes. Google Firestore, a flexible NoSQL document database, is employed for robust data storage, retrieval, and real-time synchronization, essential for handling the extensive data involved in T1D management, including physiological metrics from wearable devices and glucose levels from CGM systems (Figure 1).

### 5.2 User workflow

1. User authentication:
  - Users begin by authenticating through the WHOOP API within the React Native app. This process is handled securely using the OAuth Authorization Code Flow, ensuring that user credentials are protected throughout the authentication phase.
  - The backend server processes this authentication, fetching access and refresh tokens along with user details, which are then relayed back to the mobile application.
2. Generating custom JWT token:
  - A custom JWT token is generated for accessing Google Firestore. This token is created after verifying the user’s access token, providing a secure layer for subsequent data transactions.
3. Connecting WHOOP fitness tracker:
  - Upon successful authentication, users connect their WHOOP Health Tracker to the app via Bluetooth. This connection allows for the capture and storage of live health data such as heart rate directly into Firestore.
  - The app also ensures that all necessary permissions are obtained from the user to enable the continuous capture of this vital health data.
4. Dexcom integration:
  - Users who opt to connect their Dexcom CGM devices are guided through a secure OAuth 2.0 authorization flow using the Dexcom Developer API platform. After consent, the app obtains an access token and a refresh token that allow it to retrieve glucose data on behalf of the user.
  - The backend securely stores and manages token lifecycles. It uses these credentials to periodically query endpoints such as /v2/users/self/egvs to obtain estimated glucose values (EGVs), which represent real-time blood glucose readings at 5 min intervals.
  - Each data entry includes a timestamp, trend direction (e.g., increasing, decreasing, steady), and the glucose value in mg/dL. These data points are then pushed to Google Firestore with UTC timestamps and user IDs, allowing for precise correlation with behavioral data from WHOOP and in-app actions.
  - To preserve user privacy and ensure data integrity, all transmissions are encrypted using TLS, and access to Dexcom data is strictly scoped to glucose read permissions only. Users can revoke permissions at any time through Dexcom’s data consent dashboard.
  - Future expansions may include support for event annotations (e.g., meals, insulin doses) and retrospective trend summaries for use in the SlayBot assistant.
5. Periodic data sync:
  - The application periodically pulls additional health metrics such as sleep cycles, stress, and recovery metrics from the WHOOP servers.
  - This data is then stored and synchronized in Firestore, ensuring that users have access to the most current and comprehensive data to manage their T1D effectively.

The integration of these advanced technologies and frameworks into the Sugar Slay app not only supports the real-time functionality required for effective diabetes management but also enhances the user experience by providing a seamless, secure, and engaging interface. This technological foundation is crucial for the successful deployment and operation of the gamified decision support tool, aligning with our goals to transform T1D management into a more approachable and motivating part of everyday life.

## 6 Machine learning approach

### 6.1 Description of ML models

Our study considered various deep learning models to predict blood glucose levels for each subject. The details of these models are described below.

#### 6.1.1 Long short-term memory (LSTM) networks

Long Short-Term Memory (LSTM) networks (48), a subclass of recurrent neural networks (RNNs) (49), are adept at learning long-term dependencies, making them particularly suitable for time-series data. Two LSTM models were developed: one trained on 5 min interval data and another on 30 min interval data. Both models utilized all available features at time t to predict the glucose value at time t+1. Data was normalized using the MinMaxScaler. Each LSTM model consisted of multiple layers including 128 LSTM units, followed by sequential dense layers and dropout layers to mitigate overfitting. The architecture concluded with a final prediction layer. We employed the Rectified Linear Units (ReLU) activation function and the Adam optimizer, using mean squared error (MSE) as the loss metric, subsequently converted to root mean squared error (RMSE). Each model underwent 200 training epochs with a batch size of 32.

#### 6.1.2 Bi-directional long short-term memory (BiLSTM) networks

A Bi-directional LSTM (BiLSTM) model (50) was implemented, enhancing the model’s ability to assimilate information from both past and future contexts within the sequence. The BiLSTM shared the same architectural framework as the standard LSTM model but incorporated a bidirectional layer. Training parameters such as epochs and batch size were consistent with the LSTM models.

#### 6.1.3 Temporal convolutional networks (TCN)

Temporal Convolutional Networks (TCN) (51) excel in capturing high-level temporal relationships within sequences, making them highly effective for datasets exhibiting routine or cyclic patterns. The TCN model adopted a simpler architecture relative to the LSTM and BiLSTM, focusing on a singular TCN layer connected to a final output dense layer. The model was trained using the Adam optimizer and MSE for loss calculations, with a shorter training regimen of 10 epochs.

#### 6.1.4 Convolutional LSTM (ConvLSTM)

The Convolutional LSTM (ConvLSTM) (52) model effectively captures spatio-temporal correlations, outperforming the Fully Connected LSTM networks in many scenarios. For our application, the pre-processed data was reshaped and fed into a convolutional layer consisting of 32 filters with a kernel size of 1, followed by an LSTM layer with 128 units, and multiple dense layers. The final layer was a dense layer with 1 unit. The training involved the Adam optimizer, and the loss was measured using MSE, transformed into RMSE. Training was conducted over 200 epochs with a batch size of 32.

#### 6.1.5 Description of sequence-to-sequence models

Sequence-to-sequence (Seq2Seq) models (53), originally developed by Google in 2014, are designed to map fixed-length inputs to fixed-length outputs, even when the lengths of the input and output sequences differ. Unlike the single-step models described above (LSTM, BiLSTM, TCN, ConvLSTM), which predict only one future glucose value at a time, Seq2Seq models generate an entire sequence of future values simultaneously. This makes them particularly well-suited for multi-step glucose forecasting, where predicting trends over a 30 or 60 min horizon is more clinically useful than a single next-step prediction. These models are composed of three key components:

- **Encoder:** Features several LSTM units stacked together. Each unit processes one element of the input sequence, extracts information, and passes it on to the next unit.
- **Encoded vector:** Acts as both the final hidden layer of the encoder and the initial hidden layer of the decoder, encapsulating all the information from the input sequence.
- **Decoder:** Made up of several recurrent units. Each unit receives a hidden state as input and outputs a prediction for the corresponding time step.

Our implementation explored three variants of Seq2Seq models: Seq2Seq LSTM, Seq2Seq Bi-LSTM, and Seq2Seq CNN-LSTM. Data was segmented into 60 min windows, facilitating predictions of blood glucose levels (BGLP) up to one hour ahead, as illustrated in Table 1. For model evaluation, we employed a walk-forward validation strategy.

**Table 1:** Input and prediction mapping for 60 min data intervals.

Input	Prediction
1st 60 min data	2nd 60 min data
[1st + 2nd] 60 min data	3rd 60 min data
[1st + 2nd + 3rd] 60 min data	4th 60 min data

##### 6.1.5.1 Sequence-to-sequence LSTM

The Seq2Seq LSTM model utilized 200 LSTM cells in both encoder and decoder configurations, followed by two time-distributed dense layers with 150 and 1 units, respectively. The model underwent 80 training epochs with a batch size of 40, utilizing the Adam optimizer with a learning rate of 0.01 and mean squared error (MSE) as the loss function.

##### 6.1.5.2 Sequence-to-sequence BiLSTM

The Seq2Seq Bi-directional Long Short-Term Memory (BiLSTM) model enhances the model’s capacity to learn from data sequences with important context in both forward and reverse directions. This model comprises an encoder and a decoder, both utilizing BiLSTM layers.

- **Encoder:** Processes the input sequence and compresses the information into a context vector, summarizing the entire sequence. The forward and backward hidden states are computed as shown in Equations 1 and 2, respectively.
  $${\vec{h}}_{t}=f({\vec{W}}_{xh}x_{t}+{\vec{W}}_{hh}{\vec{h}}_{t-1}+{\vec{b}}_{h})$$ (1)
  $${\overset{\leftarrow }{h}}_{t}=f({\overset{\leftarrow }{W}}_{xh}x_{t}+{\overset{\leftarrow }{W}}_{hh}{\overset{\leftarrow }{h}}_{t+1}+{\overset{\leftarrow }{b}}_{h})$$ (2)
- **Decoder:** Generates the output sequence from the encoded state. At each time step t, the decoder’s BiLSTM layers take the previous output $y_{t-1}$ and the previous hidden states ${\vec{h}}_{t-1}$ and ${\overset{\leftarrow }{h}}_{t+1}$ to generate the current hidden state $h_{t}$ and the output $y_{t}$, as given in Equation 3.
  $$y_{t}=g(W_{hy}h_{t}+b_{y})$$ (3)

##### 6.1.5.3 Sequence-to-sequence CNN-LSTM

This model combined convolutional layers with LSTM. The encoder included two 1D convolutional layers with 128 and 64 filters, followed by a MaxPooling 1D layer and a flattening layer. The decoder used 200 LSTM cells.

#### 6.1.6 Transfer learning

Transfer learning involved identifying key features common to all subjects using a Gradient Boosting algorithm. We selected features with a cumulative importance of 0.99, focusing on attributes like finger stick, basal rate, galvanic skin response, skin temperature, and bolus dose values. We initially trained models on a randomly selected subject (#567) and fine-tuned them on individual subjects using the identified features. The configuration for each Seq2Seq model remained consistent during fine-tuning.

## 7 Results

This section presents the results from our machine learning model evaluation study using the OhioT1DM dataset. We systematically compared multiple deep learning architectures to identify the best performing model for glucose prediction in the Sugar Slay application. To contextualize the error metrics reported throughout this section: Root Mean Square Error (RMSE) quantifies the average magnitude of prediction errors while giving greater weight to large deviations, making it particularly sensitive to dangerous prediction failures in glucose forecasting. Mean Absolute Error (MAE) provides a complementary measure of average error magnitude without emphasizing outliers. Prior glucose forecasting studies report RMSE values in the range of approximately 18–22 mg/dL for 30 min predictions and around 30–40 mg/dL for 60 min horizons, with corresponding MAE values typically ranging from 12 to 20 mg/dL for shorter horizons and increasing with longer prediction windows (54, 55). These benchmarks provide context for interpreting the results presented below.

### 7.1 Training standardization and convergence criteria

All models were trained using a consistent optimization framework to ensure fair comparison across architectures. Specifically, all models employed the Adam optimizer, mean squared error (MSE) loss, identical batch sizes, and the same walk-forward validation protocol. Differences in epoch counts were not manually imposed but resulted from an early stopping strategy based on validation loss. A fixed patience parameter was applied across models, allowing training to terminate automatically once validation performance plateaued. As a result, models that converged faster required fewer epochs, whereas more complex architectures required additional iterations before stabilization. This strategy prevented overfitting and ensured that model comparison reflected architectural differences rather than unequal optimization effort. Key hyperparameters for all models are summarized in Table 2.

**Table 2:** Summary of key hyperparameters used for model comparison. Reported epoch counts indicate the maximum training budget; early stopping (fixed patience across models) was used to terminate training when validation loss no longer improved.

Model	Input granularity	Horizon	Optimizer	Loss	Batch size/Max epochs
LSTM	5 min/30 min	1-step	Adam	MSE	32/200
BiLSTM	5 min/30 min	1-step	Adam	MSE	32/200
TCN	30 min	1-step	Adam	MSE	(as used)/10
ConvLSTM	30 min	1-step	Adam	MSE	32/200
Seq2Seq LSTM	60 min windows	60 min	Adam	MSE	40/80
Seq2Seq BiLSTM	60 min windows	60 min	Adam	MSE	(as used)/(as used)
Seq2Seq CNN-LSTM	60 min windows	60 min	Adam	MSE	(as used)/(as used)

### 7.2 Model performance comparison

**Figure 2:**
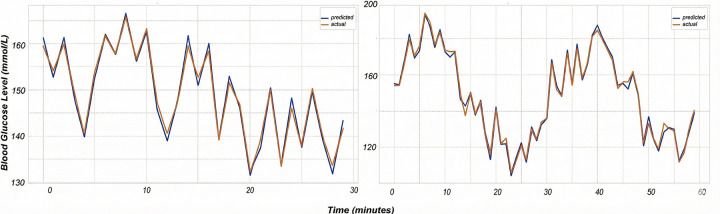
Evaluation of glucose forecasting accuracy. Comparative analysis of actual (orange) vs. predicted (blue) blood glucose levels for test subject 552 using the Sequence-to-Sequence BiLSTM architecture. The plots demonstrate the model’s predictive capability across two distinct time horizons: (Left) 30 min forecast and (Right) 60 min forecast. The close tracking of the predicted values against the ground truth highlights the model’s effectiveness in capturing temporal glucose dynamics and trend reversals.

Our comprehensive evaluation revealed that the sequence-to-sequence BiLSTM model consistently achieved the best predictive performance across all test subjects. Figure 2 illustrates the model’s ability to accurately track glucose trends for subject 552 at both 30 min and 60 min prediction horizons, demonstrating the model’s capability to capture both short-term fluctuations and longer-term patterns.

### 7.3 Best model performance metrics

Table 3 presents the RMSE and MAE values for the Seq2Seq BiLSTM model across all test subjects. The model achieved RMSE values ranging from 18.2 to 29.7 mg/dL for 30 min predictions, with corresponding MAE values between 12.7 and 18.1 mg/dL. For 60 min predictions, RMSE ranged from 28.3 to 42.6 mg/dL with MAE between 19.3 and 31.0 mg/dL. These values fall within the ranges commonly reported in the glucose forecasting literature and indicate clinically acceptable prediction performance for decision support applications (54, 55). An aggregate summary of performance across both horizons is provided in Table 4.

**Table 3:** RMSE and MAE results of the Sequence-to-Sequence BiLSTM model (best performing model).

Subjects	RMSE		MAE	
	30 min	60 min	30 min	60 min
540	24.3	41.4	17.8	31.0
544	19.8	32.9	13.7	23.1
552	18.2	30.0	13.3	22.2
567	20.7	35.1	14.4	24.9
584	29.7	42.6	18.1	30.0
596	18.6	28.3	12.7	19.3

**Table 4:** Summary of prediction performance for Seq2Seq BiLSTM across both horizons.

Metric	30 min (mg/dL)	60 min (mg/dL)
RMSE Range	18.2 to 29.7	28.3 to 42.6
MAE Range	12.7 to 18.1	19.3 to 31.0
Mean RMSE	21.9	35.0
Mean MAE	15.0	25.1

### 7.4 Comparative analysis of model architectures

To understand the superiority of the Seq2Seq BiLSTM model, we compared its performance against other deep learning architectures. Table 5 shows the RMSE values for individual single-step models at 30 min prediction horizons. While models like TCN and ConvLSTM showed competitive performance for some subjects, they lacked the consistency of the Seq2Seq models. Notably, the TCN produced an anomalous RMSE of 80.67 mg/dL for subject 544, indicating instability on certain subjects, whereas the other architectures maintained more predictable error ranges.

**Table 5:** RMSE values for individual single-step models at 30 min horizon.

Subjects	LSTM	BiLSTM	TCN	ConvLSTM
540	30.21	31.72	25.94	27.35
544	21.34	22.06	80.67	20.94
552	20.62	20.30	17.14	17.73
567	25.65	27.04	25.71	26.04
584	27.97	29.06	26.55	26.57
596	19.47	20.30	18.95	21.08

Table 6 compares the three Seq2Seq variants. Unlike the single-step models above, these architectures generate multi-step forecasts over a 60 min window, making direct comparison with Table 5 inappropriate due to the fundamentally different prediction tasks. Among the Seq2Seq variants, the BiLSTM configuration demonstrated the most consistent performance across subjects: it achieved the lowest or near-lowest RMSE for 5 of 6 subjects, and critically, its worst-case RMSE (29.7 mg/dL for subject 584) was lower than the worst-case RMSE of both the Seq2Seq LSTM (30.7 mg/dL) and the Seq2Seq CNN-LSTM (31.0 mg/dL). This consistency across subjects, rather than a single best-case result, was the primary criterion for selecting the Seq2Seq BiLSTM for integration into the Sugar Slay application.

**Table 6:** RMSE values for sequence-to-sequence models at 30 min horizon.

Subjects	Seq2Seq LSTM	Seq2Seq BiLSTM	Seq2Seq CNN-LSTM
540	25.0	24.3	23.1
544	18.5	19.8	19.2
552	19.2	18.1	17.2
567	29.2	20.7	29.0
584	30.7	29.7	31.0
596	18.6	18.6	19.0

### 7.5 Subject variability analysis

The results revealed significant inter-subject variability in model performance. Subject 584 exhibited more glycemic variability with higher peaks and lower troughs, resulting in the highest RMSE values. In contrast, subject 552 showed more stable glucose patterns, leading to the lowest RMSE values. This variability underscores the importance of developing personalization strategies for real-world deployment, where models would be fine-tuned using individual user data from their personal CGM and wearable devices.

### 7.6 Clinical interpretability and future safety evaluation

While the RMSE and MAE metrics reported above quantify overall prediction accuracy, they do not fully characterize clinical safety. Equivalent numerical errors may carry very different clinical implications depending on the glucose range in which they occur. For example, a 20 mg/dL prediction error during hypoglycemia poses a substantially greater safety risk than the same magnitude error during euglycemia. Stratified analysis of prediction performance across glycemic ranges (hypoglycemia, euglycemia, hyperglycemia) would provide valuable insight into the model’s clinical safety profile. However, as documented in prior work using the OhioT1DM dataset, there is a significant imbalance in glucose data distribution across hypoglycemic, euglycemic, and hyperglycemic ranges, with hypoglycemic events being particularly sparse (56). This class imbalance is a well-recognized challenge in glucose forecasting, as the scarcity of hypoglycemic events limits the ability of models to learn these patterns effectively and renders stratified error metrics unreliable for small subgroups (57). This limitation, combined with the fact that the dataset was collected using older sensor technology from an adult cohort that does not match our target adolescent and young adult population, makes it an inappropriate basis for drawing clinical safety conclusions. Formal clinical safety evaluation, including stratified error analysis by glycemic range and established clinical risk-grid frameworks such as Clarke Error Grid Analysis (CEGA) and the Surveillance Error Grid (SEG) (58), will be conducted in future prospective studies using data collected directly from the deployed Sugar Slay ecosystem with the target user demographic and current-generation sensor hardware.

### 7.7 Summary of machine learning results

Comprehensive quantitative benchmarking across LSTM, BiLSTM, TCN, ConvLSTM, and sequence-to-sequence variants was performed using 30 min and 60 min prediction horizons. RMSE and MAE values for all models are presented in the corresponding results tables, supporting the selection of the Seq2Seq BiLSTM architecture as the best-performing model.

The sequence-to-sequence BiLSTM model emerged as the best-performing choice for glucose prediction in the Sugar Slay application, demonstrating:

- Consistent performance across all test subjects
- Clinically acceptable error rates for both 30 min and 60 min predictions (59)
- Superior ability to capture bidirectional temporal dependencies in glucose data
- Robust performance without requiring complex transfer learning approaches

These results provide strong evidence for integrating the Seq2Seq BiLSTM model into the Sugar Slay application, where it will work in conjunction with real-time data from WHOOP and Dexcom G6 devices to provide personalized glucose predictions for users.

The OhioT1DM dataset was used exclusively for model benchmarking and architecture comparison. Real-time WHOOP and Dexcom integrations are presented to demonstrate deployment feasibility and were not used for model training within this study.

## 8 Prototype development of gamified decision support tool for T1D management

### 8.1 Concept and design

**Figure 3:**
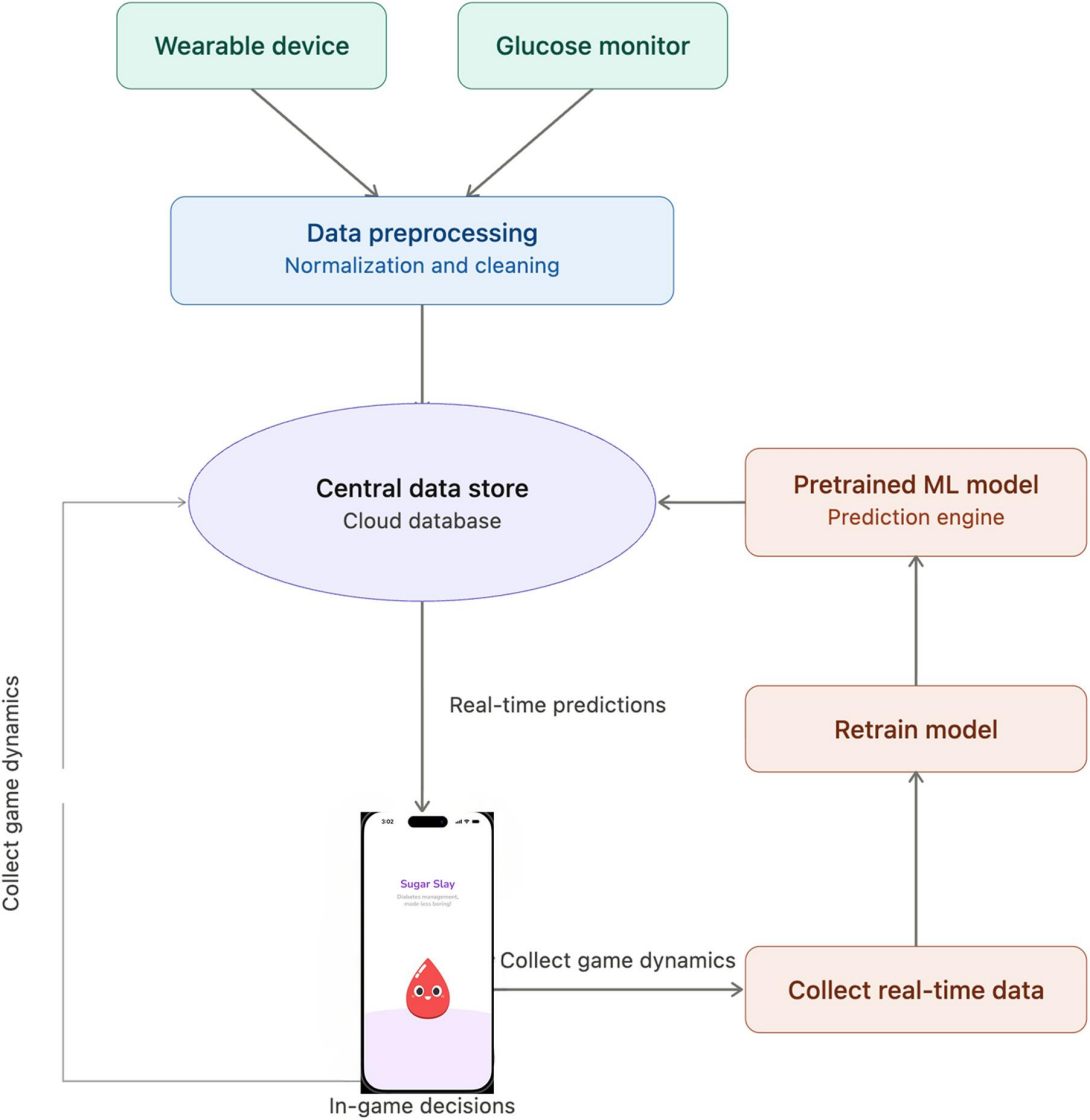
System architecture and data flow of the Sugar Slay platform. The framework integrates physiological data streams from Continuous Glucose Monitors (CGM) and wearable sensors to facilitate real-time preprocessing and normalization. Data is synchronized via Cloud Firestore, which serves as the central hub connecting the user interface with the machine learning pipeline responsible for model inference and periodic retraining. The mobile application delivers real-time glucose predictions to the user while simultaneously capturing in-game decisions and game dynamics to close the feedback loop.

Building on the advanced integration of Continuous Glucose Monitoring (CGM) and physiological data from wearable technologies discussed earlier, our research developed a novel, gamified decision support tool through a structured and iterative design process. The overarching objective of this system is to transform routine T1D management from a clinical necessity into an engaging, motivational experience that encourages sustained user engagement. To facilitate this shift, the application employs a comprehensive gamification framework that includes tailored challenges, achievement badges, detailed progress tracking, and social features. These elements are specifically designed to make daily diabetes management tasks feel inherently more rewarding and significantly less burdensome for the user. The specific gamification dynamics were grounded in insights from early-stage user interviews and feedback collected during the initial design phase, with subsequent refinements made based on empirical results from usability testing. We conducted a specific usability study with 7 participants with T1D to evaluate the app’s features, interface design, and the effectiveness of these gamification elements. The proposed app architecture, illustrated in Figure 3, reflects an interactive ecosystem that directly links user behavior to diabetes management goals by actively engaging gamification mechanics. Key findings from our study indicated that users deeply appreciated the integration of health metrics with game mechanics, demonstrating strong user acceptance of the app’s design philosophy and feature set.

### 8.2 Key usability study findings

Participants demonstrated strong engagement with the core application features, particularly the Data Logging, which 100% of users found “very easy” for meals (Table 7). While the gamification elements were effective, with 71% finding challenges motivating, feedback suggested that the initial display was overwhelming. Users recommended a progressive unlocking system and difficulty tiers to improve the experience. The Glucose Forecast was highly valued as a novel feature, with 71% of participants stating they would use it daily, though requests were made for clearer explanations of the prediction confidence ranges. Finally, the Social Groups feature generated the highest perceived value (83%), with participants specifically requesting the addition of message boards to facilitate peer support.

**Table 7:** Quantitative usability metrics by feature (*N* = 7).

Feature	Key user metrics
Onboarding process	57% found it “mostly” helpful
	57% found it “mostly clear”
	57% “mostly confident” to continue
	71% rated “very easy” to understand
Glucose forecast	71% “very likely” to use daily
	57% found range presentation “very helpful”
	100% rated meal logging “very easy”
Data logging	83% rated mood logging “very easy”
	71% rated insulin logging “very easy”
Insights	86% found navigation “very easy”
	43% found insights directly “helpful” (mixed understanding)
Challenges	71% found them “motivating”
	57% “likely” to return specifically for challenges
Badges	100% understood the badge system well
	50% found the specific badges “meaningful”
Groups	83% believed groups would be “very valuable”
	83% felt “very comfortable” joining
	67% were “very interested” in joining

### 8.3 Prototype features

Based on our usability study findings, we refined the following features:

1. **Progress tracking and achievements:** Users engage with gamification elements including challenges and achievements. Nearly all participants (71%) found the challenges to be “very motivating” or “motivating,” with feedback suggesting that visual rewards and progress tracking enhanced engagement.
2. **Challenges and rewards:** Tasks such as managing glucose levels, adhering to diet, and maintaining physical activity are framed as challenges within the app. The usability study revealed that users particularly appreciated the XP system and the ability to track streaks, with many requesting additional customization options like “skins” similar to popular games.
3. **Supportive companion features:** The app includes supportive, encouraging elements that provide non-judgmental feedback and motivation. Users expressed interest in having companion-like features that offer emotional support and celebrate their achievements without the clinical feel of traditional diabetes management tools.
4. **Integration of real-time data:** CGMs and wearables like the WHOOP band feed data to provide real-time insights. Over 71% of participants rated the glucose forecast feature as “very easy” to understand and were “very likely” to use it daily, validating its importance in the app.
5. **Gamification elements:** The study confirmed strong interest in social features, with 67% “very interested” in joining groups and 83% believing group interaction would be “very valuable.” Users specifically requested message board functionality and clearer group challenge mechanics.

### 8.4 Sugar slay care: needs assessment for companion support

**Figure 4:**
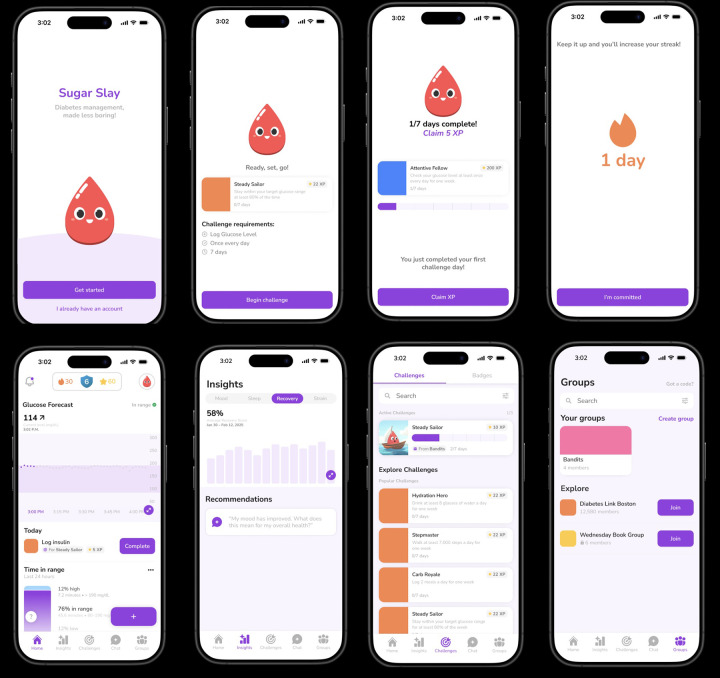
Key interface modules of the Sugar Slay application. The user experience combines clinical data management with immersive gamification to promote sustained engagement. The top row illustrates core tracking features, including real-time glucose visualization, forecasting, and multimodal data logging. The bottom row depicts the gamification ecosystem, showcasing avatar customization, achievement badges, social leaderboards, and streak-based rewards designed to reinforce positive health behaviors.

During the usability sessions for the main Sugar Slay application (Figure 4), a recurring theme emerged regarding the social nature of diabetes care. Participants frequently mentioned that their management strategies heavily relied on, or were complicated by, interactions with family and friends. Based on these interviews, we discovered a distinct need for a companion application that could bridge the communication gap between patients and their support network. While the primary app empowers the individual, caregivers often reported feeling anxious or unsure how to help without overstepping.

To support this holistic ecosystem of T1D management and validate these insights, we conducted a targeted qualitative need-finding study with 6 participants who support individuals with T1D. This cohort included parents, siblings, and friends, providing diverse perspectives on the challenges of being a “supporter” vs. a “manager” (Table 8).

**Table 8:** Need-finding analysis for *Sugar Slay Care* (*N* = 6).

Participant	Primary need/anxiety	Key design implication
P1 (Sibling)	Safety during isolation (e.g., solo runs).	“Nudge” button for low-friction check-ins.
P2 (Friend)	Guidance on how to offer support.	Milestone notifications and support tips.
P3 (Parent)	Fear of sleeping through nighttime lows.	Emergency alerts that bypass phone silence.
P4 (Parent)	Overwhelmed by raw data streams.	“Status at a glance” dashboard (2–3 metrics).
P5 (Parent)	Managing logistics and mood impacts.	Prescription tracking and mood/glucose overlays.
P6 (Parent)	Understanding context behind trends.	Weekly retrospective summaries and event tags.

The interviews revealed several critical themes that guide the design of *Sugar Slay Care*:

- **Safety vs. Autonomy:** A recurring theme, particularly among parents of young adults (aged 16–18+), was the tension between ensuring safety (e.g., night-time lows) and respecting the growing independence of the individual. As shown in Table 8, parents of older children preferred “alerts only” or weekly summaries rather than constant surveillance.
- **Actionable insights over raw data:** Supporters often felt overwhelmed by raw numbers. They expressed a strong preference for correlated data, specifically understanding *why* a spike occurred (e.g., “high stress” or “poor sleep”) rather than just seeing the number. One parent noted the need for a “status at a glance” dashboard that synthesized complex metrics into simple actionable statuses.
- **The “Nudge” Mechanism:** Supporters wanted ways to intervene that didn’t feel like “nagging.” Features like a “nudge button” or gentle automated reminders were highly requested to maintain positive relationships while ensuring health tasks were completed.
- **Emergency protocols:** For high-risk scenarios (e.g., severe lows at night or during solo exercise), supporters requested robust alerting features that could bypass phone silence settings, similar to “Find My iPhone” emergency pings.

**Figure 5:**
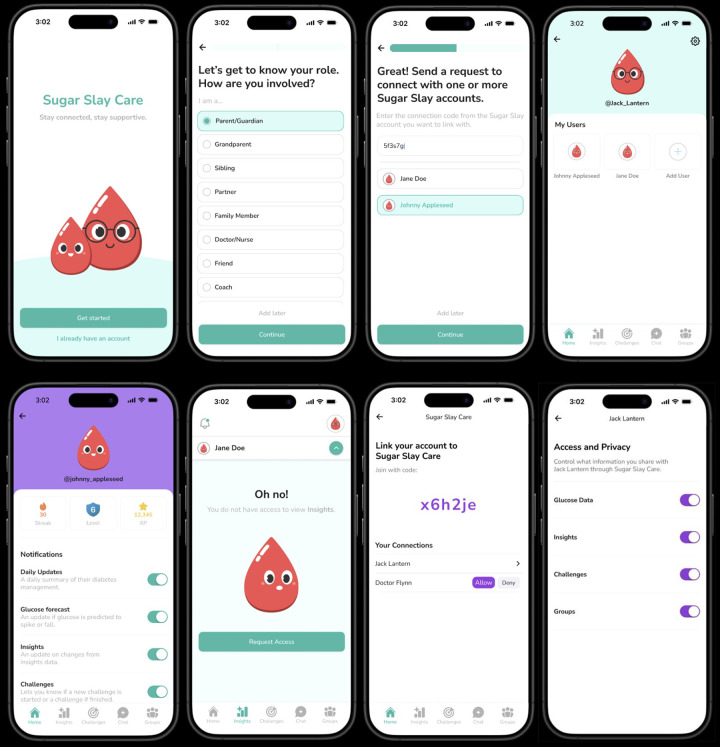
Interface design of the Sugar Slay Care companion application. Geared towards the support network, this module emphasizes role-based access control and granular privacy settings. The screens demonstrate the onboarding workflow where caregivers identify their relationship to the patient (e.g., parent or partner) and the secure pairing process using unique connection codes. The interface prioritizes user autonomy by allowing the primary user to toggle specific data permissions, ensuring caregivers receive actionable insights while respecting patient privacy boundaries.

Based on these findings, we designed *Sugar Slay Care* to offer customizable permission levels that adapt to the age and autonomy of the user, ensuring that support remains helpful rather than intrusive. The interface design of the companion application is shown in Figure 5.

### 8.5 Implementation strategy

Building on the insights gained from our usability study and the architectural framework defined earlier, the transition from prototype to a fully functional deployment involves a multi-phased implementation strategy. This phase focuses on integrating the validated machine learning models with the gamified user interface while ensuring robust data security and privacy.

#### 8.5.1 Data handling and security

The foundation of the Sugar Slay ecosystem relies on the seamless integration of physiological data. The app will ingest real-time streams from Continuous Glucose Monitors (CGM) and wearable sensors through encrypted, time-aligned APIs, as demonstrated in our technology integration section. Ensuring end-to-end encryption is paramount not only for compliance but also to maintain the trust of our users—a critical requirement identified during our need-finding interviews with caregivers.

#### 8.5.2 Machine learning integration

At the core of the decision support system is the Seq2Seq BiLSTM model, which demonstrated superior performance in our preliminary evaluations. This model will be deployed to generate the 30 and 60 min glucose forecasts. Our usability testing confirmed that users place high value on this predictive capability, with 71% stating they would use the forecast daily. The implementation will prioritize low-latency inference to ensure these predictions are available in real-time to support immediate decision-making.

#### 8.5.3 User interface refinements

Directly addressing the feedback from our usability participants, the final interface implementation will undergo specific refinements before public release. Key updates include improving visual contrast for better accessibility and providing clearer, contextual explanations for the “Insights” generated by the system. We will also tighten the integration between related features, such as linking meal logging directly with insulin bolus calculations, to streamline the user workflow and reduce friction.

#### 8.5.4 Expected outcomes

The successful execution of this strategy is expected to significantly increase user engagement and improve adherence to diabetes management protocols. By combining emotional design with intelligent decision support, we aim to empower T1D individuals to take ownership of their health. Our usability testing strongly supports this potential, with participants demonstrating a high likelihood of continued use and finding critical tasks like meal logging extremely easy. Ultimately, Figure 4 illustrates how these foundational pillars—Gamification, Groups, Personalized Insights, and Self-Discovery—converge to create a sustainable ecosystem for user engagement.

## 9 Discussion

In this study, we present Sugar Slay, an innovative platform for managing T1D developed through three complementary investigations: (1) a machine learning model evaluation to identify the best performing glucose prediction algorithm, (2) a usability study to assess user acceptance of the app’s gamification features and interface design, and (3) a qualitative need-finding assessment to define the requirements for a companion support system. By leveraging narrative engagement through gamification elements, we have designed and implemented an application that integrates real-time physiological data from wearable fitness trackers and CGMs. The app leverages the sequence-to-sequence BiLSTM model, which demonstrated the best predictive performance in our evaluation. Collectively, these studies validated the app’s design choices, underscored the importance of the social care network, and identified key areas for improvement.

### 9.1 Machine learning model performance

Our comprehensive evaluation of multiple machine learning models on the OhioT1DM dataset revealed that the sequence-to-sequence BiLSTM model consistently outperformed other approaches. This model achieved RMSE values between 18.2 and 29.7 mg/dL for 30 min predictions across different subjects, demonstrating its potential for accurate glucose forecasting. The bidirectional nature of the model allows it to capture both past and future context, which is particularly valuable for glucose prediction where trends and patterns in both directions provide important information.

### 9.2 Integration and analysis of wearable device data

In developing our pilot app for T1D decision support, we demonstrated successful extraction and tracking of physiological metrics from a wearable fitness sensor, including heart rate variability, sleep quality, and recovery metrics. Additionally, we successfully extracted and tracked data from Continuous Glucose Monitoring (CGM) systems. These data provide crucial insights into the user’s overall health and stress levels, which are known to influence blood glucose management in individuals with Type 1 Diabetes (T1D). The machine learning models trained on the OhioT1DM dataset (47) demonstrated the feasibility of predicting glucose levels effectively using historical data. While early iterations operated with independent data streams, the insights gained here were fundamental to the architecture of our final deployed application.

### 9.3 User experience and gamification dynamics

Our usability study with 7 participants provided valuable insights into user acceptance, specifically highlighting the intersection of clinical utility and gamification. The glucose forecast feature showed exceptional user acceptance, with 71% finding it “very easy” to understand and stating they were likely to use it daily. Beyond the functional utility, users responded positively to the gamification elements, validating the core premise of our design. Participants appreciated elements that provided immediate feedback through XP gains and long-term goals via badges and achievement tracking. The social aspect also resonated strongly, with 67% of users expressing strong interest in group features. These findings align with literature showing that gamification can significantly increase user interaction and adherence (37, 60).

However, the study also identified specific areas for refinement regarding visual design and feature cohesion. Participants consistently requested more visual variety, including additional colors, better contrast, and customization options like “skins” or themes to express their identity within the app. Furthermore, users pointed out the need for tighter integration between related features, suggesting that meal and insulin logging should be linked more intuitively. They also requested clearer educational explanations of how different metrics relate to glucose trends, indicating that while the data is useful, the “why” behind the data is equally important for user empowerment.

### 9.4 Overcoming data silos through unified architecture

A significant challenge identified in this study was the fragmentation of data sources. Wearable fitness tracking data and CGM data are typically siloed, managed and accessed through separate systems and applications. This separation hinders the holistic management of T1D, where physiological stress markers and glucose levels need to be simultaneously considered for optimal decision-making. To address this integration challenge, we developed a single, unified application that consolidates data from wearable fitness trackers, CGM, and the predictive insights from our ML models trained on the OhioT1DM dataset (47).

This implemented application (Figure 3) effectively bridges these gaps by not only displaying real-time data from disparate devices but also providing predictive analytics on glucose levels. Incorporating our usability findings, the platform prioritizes clear visualization of glucose predictions with explanations and seamless integration between related logging features (meals, insulin, glucose). By embedding these technical capabilities within a framework of engaging gamification—complete with meaningful rewards, social support features, and customizable themes—we have created a comprehensive tool that maintains user interest while managing the complexities of T1D.

### 9.5 Next steps: expanded user studies and clinical validation

With the application fully developed and refinements from the usability study implemented, our immediate focus shifts to expanded validation and technical personalization. We plan to conduct studies with larger, more diverse user groups to validate our findings and identify additional improvement areas across different demographics. Concurrently, we are refining the personalization algorithms within the live app environment to allow the Seq2Seq BiLSTM model to adapt to individual user patterns over time. This step is crucial for moving from a generalized model to a truly personalized decision support tool.

Once the personalization parameters are tuned, we will initiate a clinical pilot study to evaluate objective health outcomes. This study will assess key metrics such as time in range and HbA1c levels, alongside quality of life measures, to determine the clinical efficacy of the intervention. Critically, this prospective data collection phase will also enable formal clinical safety evaluation using established error grid frameworks, including Clarke Error Grid Analysis (CEGA) and the Surveillance Error Grid (SEG) (58), as well as stratified performance assessment across glycemic ranges, using data collected from the target user population with current sensor technology. This clinical risk-grid analysis was intentionally deferred from the present study, as the OhioT1DM benchmarking dataset was collected using older technology from a limited adult cohort, making it an unsuitable basis for clinical safety conclusions applicable to our adolescent and young adult target population. Finally, understanding that diabetes management is a lifelong endeavor, we will analyze user retention and engagement patterns over extended periods. This long-term analysis will help us understand which gamification features drive sustained use and prevent the attrition often seen in digital health interventions.

## 10 Conclusion

In conclusion, our study reports on the first steps toward creating a novel, personalized approach in managing Type 1 Diabetes (T1D) by integrating physiological data from wearable technologies with predictive machine learning models and engaging gamification features. We identified the sequence-to-sequence BiLSTM as the best model for glucose prediction integrated into our app. We also validated our app design through usability testing with target users. Our proposed unified application is innovative not only because it supports comprehensive decision support through accurate glucose predictions, but also due to its carefully designed gamification features that enhance user engagement and motivation. The app’s companion-like features provide supportive, non-judgmental feedback that resonates with users seeking alternatives to clinical interfaces. This approach transforms daily diabetes management tasks into engaging challenges with meaningful rewards, fostering a sense of achievement and progress. The social elements further enhance motivation through community support and shared experiences. The combination of advanced machine learning for glucose prediction with user-centered design principles and gamification elements represents a significant innovation in digital health. Our usability study findings provide a roadmap for refinements that will make the app more engaging and effective.

This work should be interpreted as an early-stage development and technical validation study. While real-time integration with Dexcom G6 and WHOOP devices was implemented at the system level, clinical deployment and longitudinal evaluation of behavioral outcomes were outside the scope of this study. The present study focuses on model benchmarking and ecosystem design; large-scale clinical validation and personalization represent important directions for future work. Future work will focus on prospective real-world evaluation including adherence, engagement, and glycemic outcomes.

While Sugar Slay is motivated by the goal of reducing cognitive burden associated with T1D management, the present work does not directly measure cognitive load or quality-of-life outcomes using validated clinical instruments. Instead, this study focuses on early-stage ecosystem development and usability exploration. Future clinical studies will incorporate validated questionnaires and longitudinal behavioral assessments to quantitatively evaluate cognitive burden reduction among users and caregivers.

If successful, this approach could serve as a model for other chronic conditions where continuous monitoring, predictive analytics, and sustained user engagement are crucial for disease management. This integration of advanced technology with engaging, motivational elements aims to transform T1D management from a burden into an empowering journey toward better health.

## 11 Limitations

This study represents an early-stage development effort and has several limitations. First, machine learning models were evaluated using the OhioT1DM dataset rather than data collected directly from the deployed ecosystem, limiting conclusions regarding real-time clinical performance. The OhioT1DM dataset, while widely used for benchmarking, was collected using older sensor technology from a limited adult cohort, which constrains the generalizability of clinical safety conclusions to our target adolescent and young adult population. Furthermore, the dataset exhibits a significant imbalance in glucose data distribution, with hypoglycemic events being particularly sparse (56, 57), which precluded robust stratified error analysis across glycemic ranges and rendered formal clinical risk-grid evaluation [e.g., Clarke Error Grid Analysis or the Surveillance Error Grid (58)] inappropriate at this stage. These analyses are deferred to future prospective studies using data from the deployed system. Second, while usability and need-finding studies provide valuable qualitative insights, the sample sizes were small and not intended for statistical generalization. Third, claims regarding cognitive burden reduction and behavioral outcomes remain hypothesis-driven and were not quantitatively validated in this work. Finally, although real device integration (WHOOP and Dexcom) was implemented, pilot deployment evaluating engagement, adherence, or glycemic outcomes has not yet been conducted.

## 12 Next steps

The development roadmap includes three sequential phases: (1) personalization refinement of glucose forecasting models using live user data, (2) pilot deployment studies evaluating engagement and feasibility, and (3) controlled clinical evaluation assessing glycemic outcomes, adherence, quality-of-life measures, and formal clinical safety evaluation including error grid analysis (CEGA and SEG) (58) using prospectively collected data from the target population. This staged approach reflects the early maturity of the current system while defining clear steps toward clinical validation.

## Data Availability

The original contributions presented in the study are included in the article/**Supplementary Material**, further inquiries can be directed to the corresponding author.

## Supplementary material

Demo Video A demo of the Sugar Slay app can be accessed at the following URL: Youtube Video A demo of the Sugar Slay Care app can be accessed at the following URL: Youtube Video
