# Supplementary material for: Sugar slay: a gamified decision support ecosystem for type 1 diabetes
Source: Front Digit Health. 2026 Jun 17;8:1779790. doi: 10.3389/fdgth.2026.1779790 (PMC13319092; doi:10.3389/fdgth.2026.1779790)
Supplement: Supplementary file 3 [file Datasheet1.pdf]

Onboarding Process:

How well did the onboarding help you understand key features?

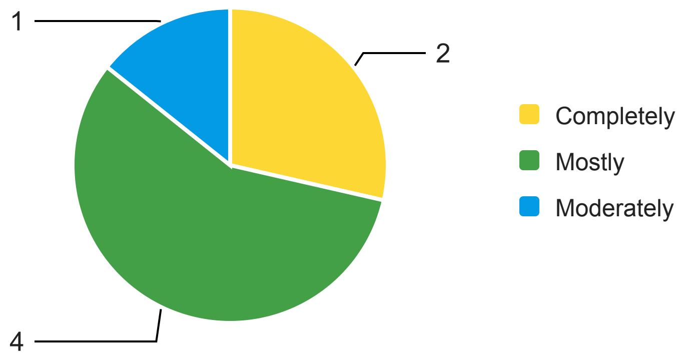

How clear was the onboarding process?

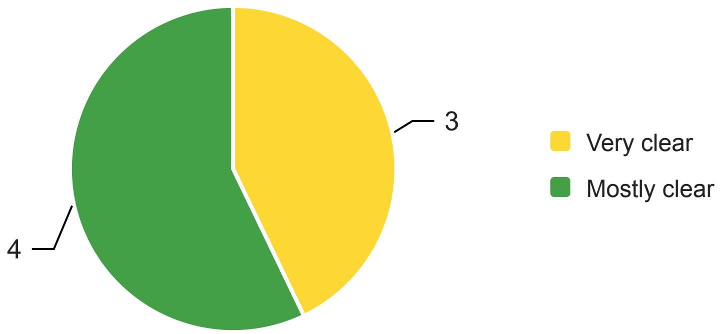

How visually engaging did you find the onboarding experience?

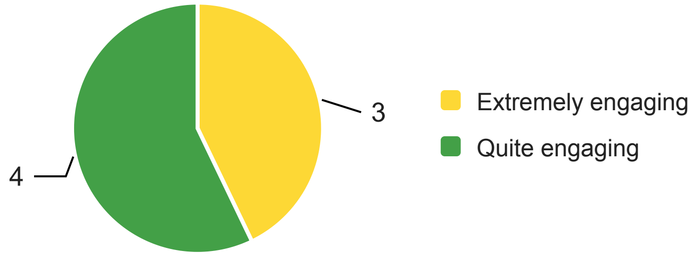

How confidently do you feel that you would be able to continue using the app without further training?

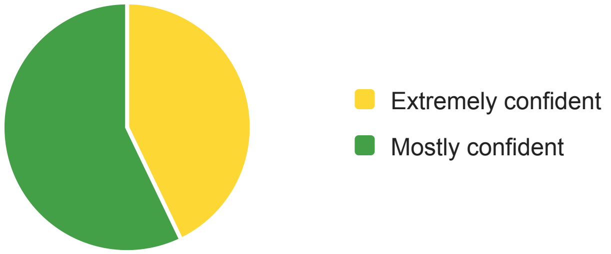

Select a Source

Glucose Forecast

Q6 - How easy is it to understand your forecasted glucose trends?

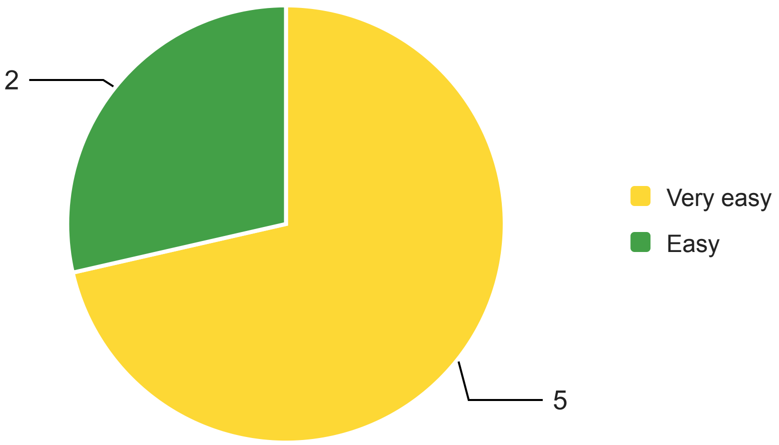

Q7 - How helpful do you find the range presentation?

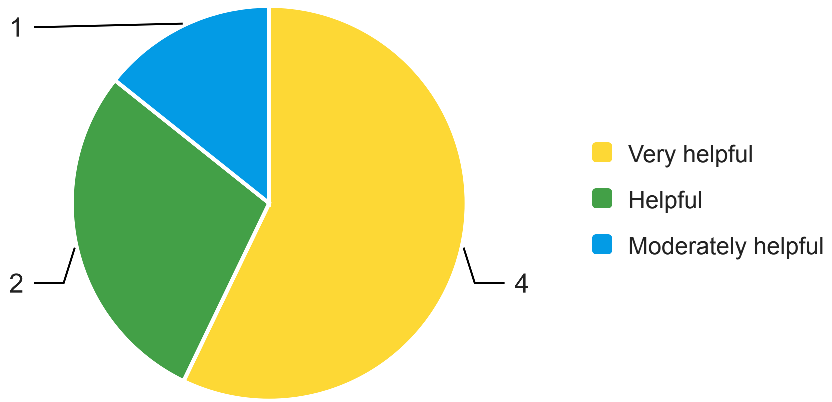

Q8 - How likely are you to use this feature daily?

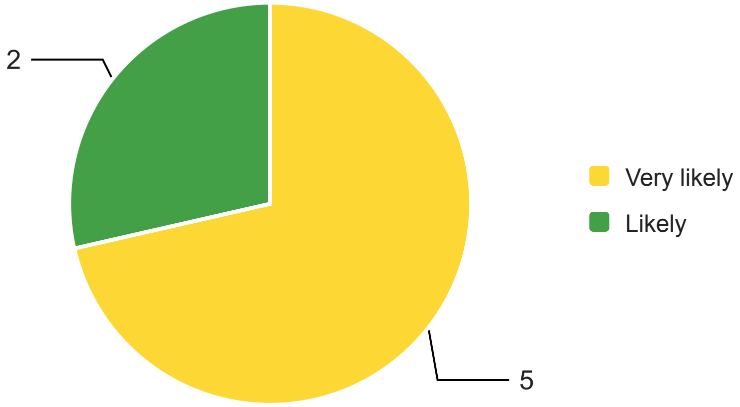

Q29 - Are there any insights we’re missing?

Are there any insights we’re missing?

It would be cool to add a notes section to the metrics themselves. Add a journal for each metric.

none that i can think of now.

Average Time in Range over time. Average insulin usage

It would be cool to see how each insight compares to my glucose level. Adding period tracking would be cool.

It would be cool to see how my blood sugar changes with these.

I guess strain kind of represents exercise. it would be cool to see a time in range for heart range. at the endocrinologist, they ask "have you been getting 180 mins of exercise a week?" they count that as your green-level heart range.

Q9 - Do you have any other thoughts on the glucose forecast

Do you have any other thoughts on the glucose forecast

It's definitely something we don't have with the Dexcom / cgm system. Dexcom doesn't show you a forecast. This is really interesting, it would be super helpful. The only thing is the range presentation, it could be made more clear. Perhaps add a border line.

the only thing i would be worried about is the legality behind it. users could hold you liable if ur forecast is incorrect.

I would look at other CGM apps. It would be helpful to see why it’s forecasted like that. What was it that I did that changed the forecast.

Does the user know how to interact with the glucose forecast?

The glucose forecast would be intuitive for Dexcom users, but perhaps not for others.

Unless u guys have a pump feature, id be moving back and forth between this and my other apps.

Cool, but Dexcom already has this. You should add a haptic when dragging between glucose dots.

Yes. Something that Dexcom does, which is super helpful, is that you can change the view.

Q14 - How easy is it to log meals?

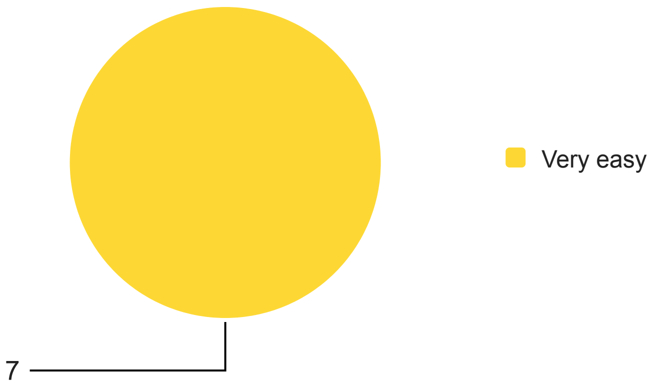

Q15 - Do you like this form of meal logging?

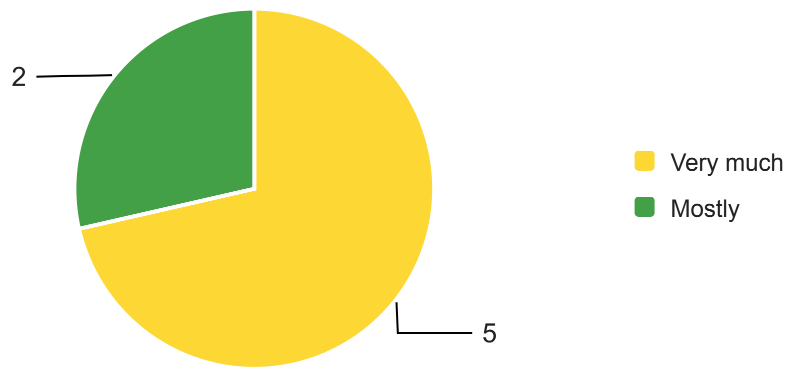

Q16 - How likely are you to log meals?

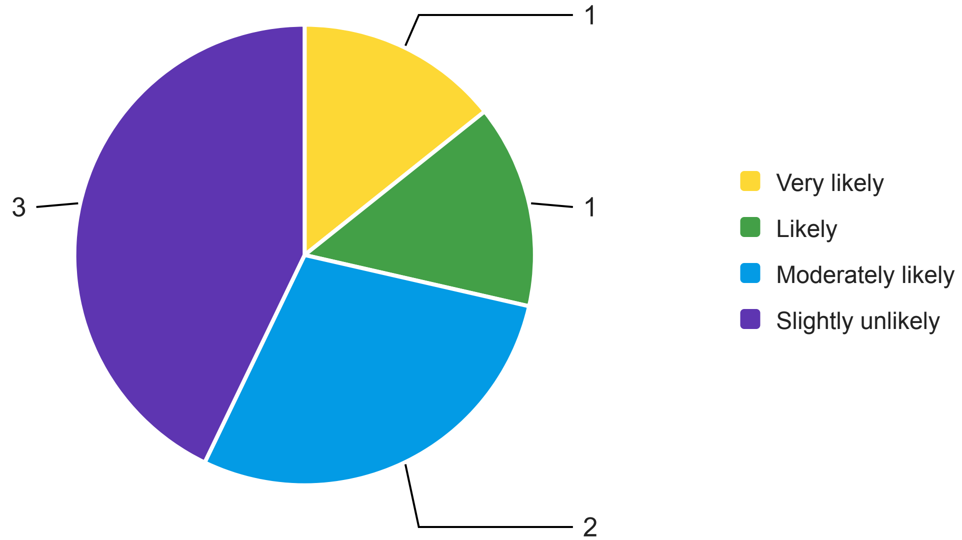

Q18 - How easy is it to log insulin?

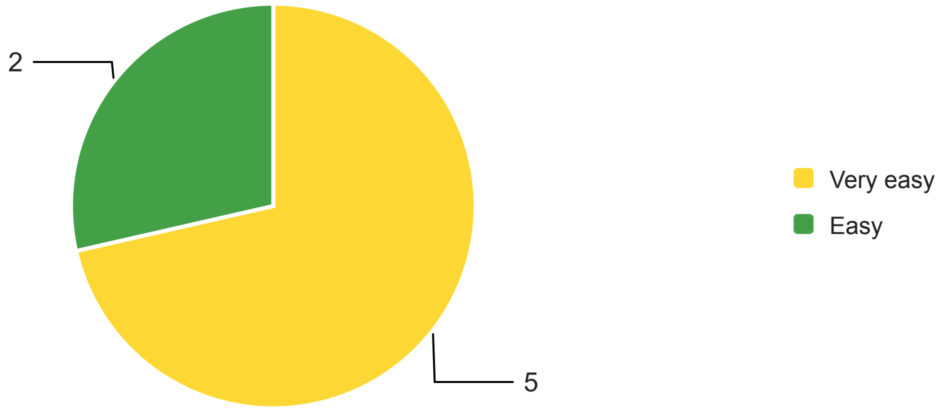

Q19 - How likely are you to log your insulin?

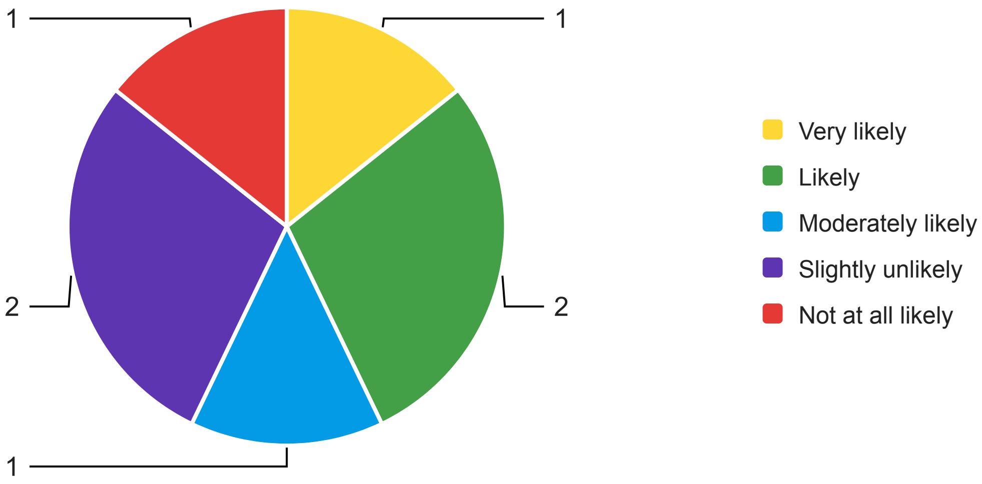

Q20 - How easy is it to log your mood?

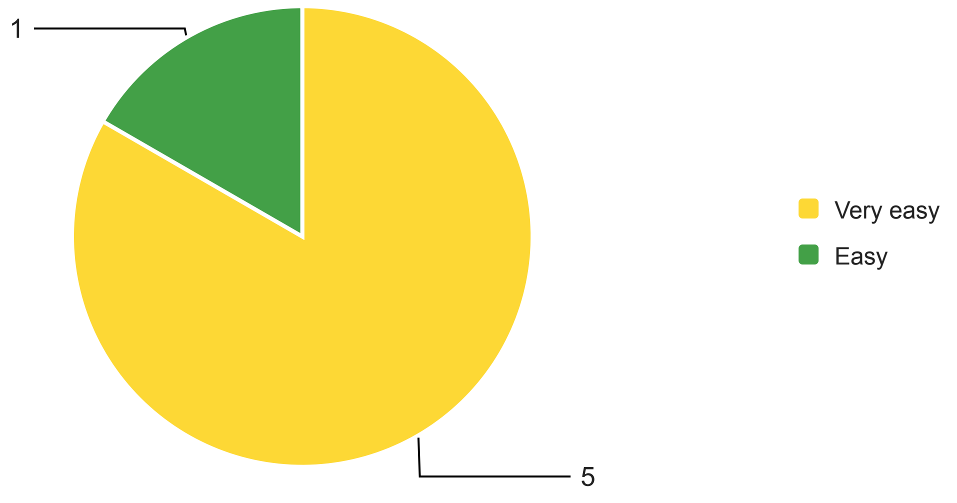

Q21 - How likely are you to log your mood?

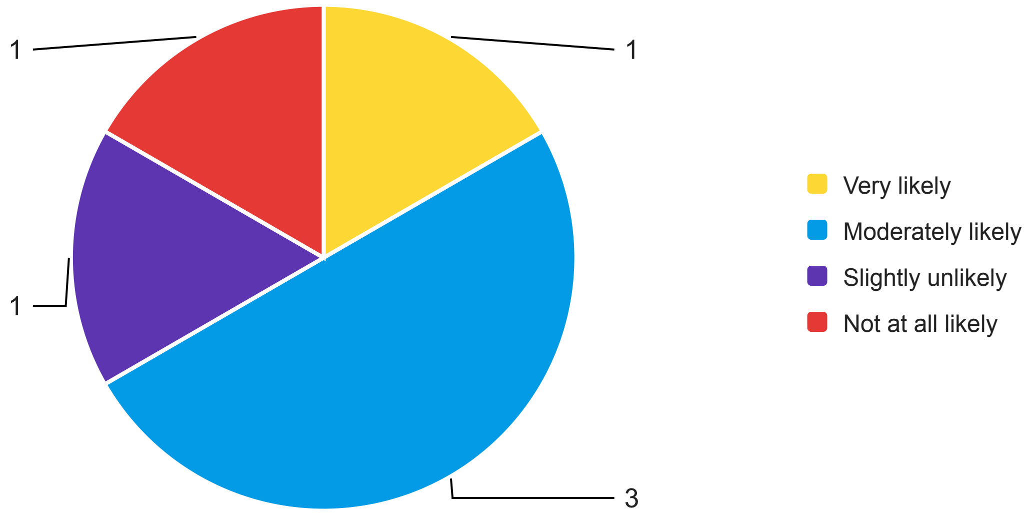

Q22 - Do you have any other thoughts on the design of logging data?

Do you have any other thoughts on the design of logging data?
For a pump user, there's no option to put in a basel rate.<br><br>I like the notes section in mood logging.
after meal logging: i like that you gain xp for logging stuff.<br>insulin: it's a bit redundant with the short acting and bolus. i like that you're using it to collect data. insulin log would make it easier to find the correlation between insulin and other metrics.<br><br>general notes: incentivize people to log their mood. perhaps give users periodic reminders to log their data. logging insulin and meals is something i do anyway. asking users to log their mood is a bit more of an ask.
It would be helpful of the meals showed up on the glucose forecast. Also would be cool to add notes about the meal. Make units consistent. For mood, it doesn’t really explain what it is. Add notes for mood. Add more information about why users should track their mood. Month in review
I can’t imagine that many people would log their insulin. If you click a number, it pops up a box and you can enter a word to describe that feeling.
Insulin logging:<br>- Confused about the difference between short acting and bolus. Are they supposed to be two different things, or are they the same?<br>- Another thing, on My Bolus in the tandem app, when i input the carbs, it shows how many units i need (5 for 50 carbs).<br>- Does this include a correction for food? Bolus vs correction bolus. Look into that. Tandem app does it well.<br>- A bolus is any method of taking insulin that counteracts ur blood sugar going up. almost always related to food. if ur high and dont have food, then it's a correction.<br><br>Meal logging:<br>- Meal logging and bolus logging are the same thing, so these might not need to be.<br>- Maybe log meal and then route users to log bolus.<br><br>Mood logging:<br>- Reminder would be cool.

Maybe add a screen that says "we wont sell you data".

Depending on whether or not ur using a pen/syringe, u can just enter the data. but if you're on a pump, you don't have to "log" your insulin. since im on a pump, id rather answer "did you bolus for lunch? option to add the units of the bolus." I'd have gone with . check out the feelings wheel for mood logging. everything has a color on the feelings wheel, so you could just add a color to each of the mood terms.

I think for newer diabetics, this is super helpful. for users who are several years out, it might not be as intriguing.

Q23 - How understandable are the insights provided

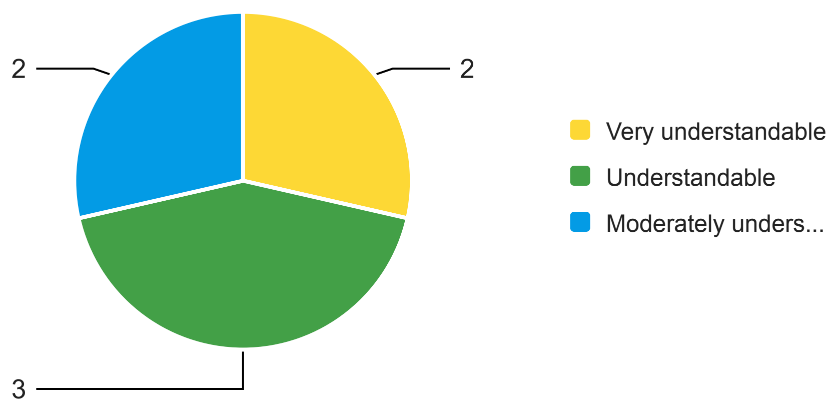

Q24 - How helpful do the insights feel?

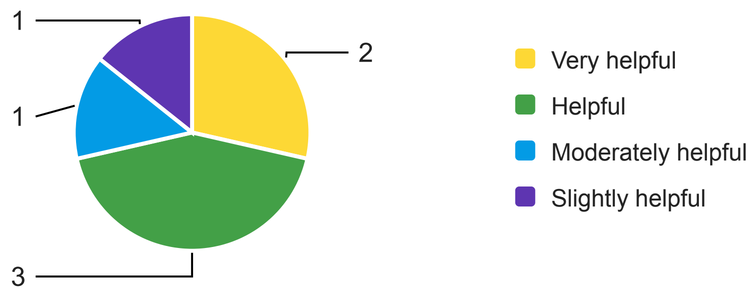

Q25 - How easy is it to navigate between insights?

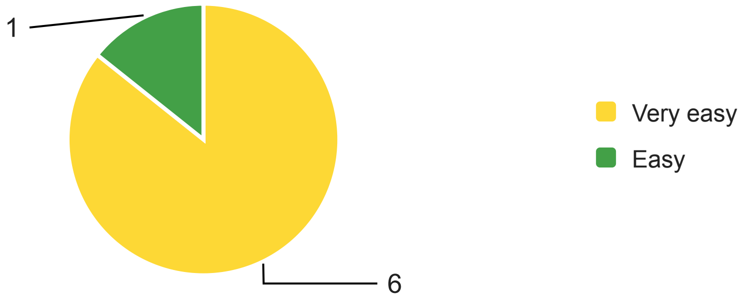

Q26 - How visually appealing do you find the data presentation?

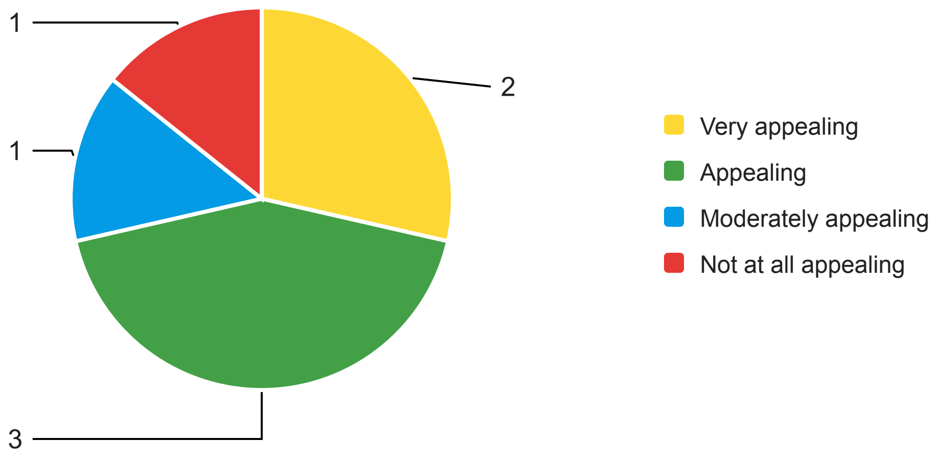

Q29 - Are there any insights we're missing?

Are there any insights we're missing?
It would be cool to add a notes section to the metrics themselves. Add a journal for each metric.
none that i can think of now.
Average Time in Range over time. Average insulin usage
It would be cool to see how each insight compares to my glucose level. Adding period tracking would be cool.

It would be cool to see how my blood sugar changes with these.

I guess strain kind of represents exercise. it would be cool to see a time in range for heart range. at the endocrinologist, they ask "have you been getting 180 mins of exercise a week?" they count that as your green-level heart range.

Q30 - Do you have any other thoughts on the insights feature?

Do you have any other thoughts on the insights feature?

recommendations should explain the correlation or actual meaning of the insights. how will this data affect me?

More colors, maybe each color has a meaning. I'd prefer the chat bot. Could the chat bot provide links. Insights: id like more recommendations and some explanation of what the data means for my health / how the data occurred. Add more colors. Log exercise. Make whoop data optional

Data presentation is a little dull. More contrast would be nice. More colors.

would be cool to have a different, more prominent color

Maybe change the colors of the bars. Something that highlights. From a kids perspective.

I guess the only reason i docked the first one a point is because they're on different scales. if they were on the same scale, it would be easier to understand.

Q31 - How easy is it to understand each challenge?

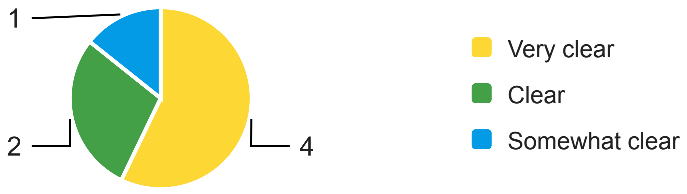

Q32 - How motivating are challenges for you?

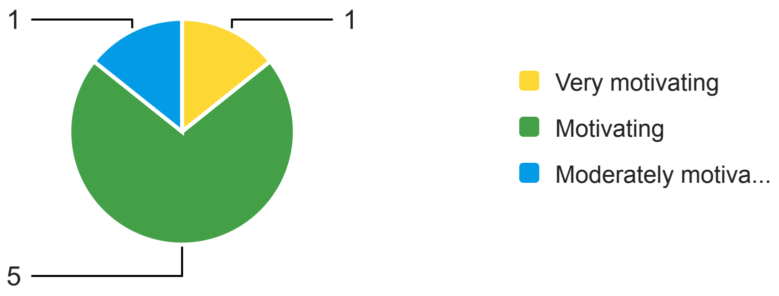

Q33 - How likely are you to return to continue a challenge?

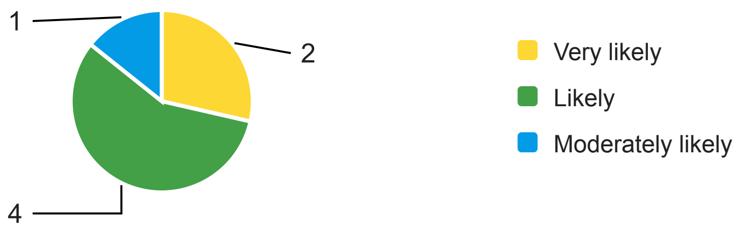

Q34 - How well do challenges fit into your daily routine?

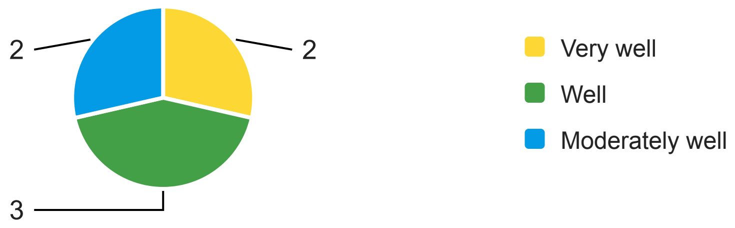

Q35 - Do you have any other thoughts on challenges?

Do you have any other thoughts on challenges?
I like the idea of challenges. Good that they'd help me lead a healthy lifestyle. I would try to rank the challenges by difficulty, with harder challenges offering more xp.
I'd like to buy skins with my xp. Would be cool if family members could high five you. More incentives, like skins in Roblox.

How the explore challenges are displayed is a lot. Maybe consider unlocking challenges as you increase in level / complete challenges. Add educational details for challenges: what’s the purpose of completing them for my health?

unique log for each challenge could be cool. log button ON the challenge itself.

the scrolling doesn't work very well. it's too sensitive.

For the challenges, i would look at Maslow's hierarchy of needs. start with something like water, food, sleep, etc. then, add in exercise or steps, or recovery. Layering in "how do you be a human before you be a diabetic".

My OCD brain does not like that 150xp points isn't divisble by 7 (7 days). make each day 50pts or something like that.

Q40 - Do you have any other thoughts on badges?

Do you have any other thoughts on badges?

badges should offer more xp. based on difficulty ofc. there should be an indication that certain badges are harder to unlock.

it was unclear how maxed out my badge is. i like the idea of getting new badges. maybe an exponential tier system: ex. bronze, silver, gold, etc.

Feels like graduating the next grade level. It feels like you've reached mastery.

Q36 - How meaningful do badges feel to you?

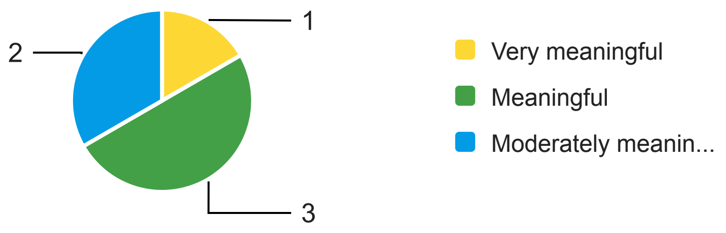

Q37 - How well do you understand what badges represent?

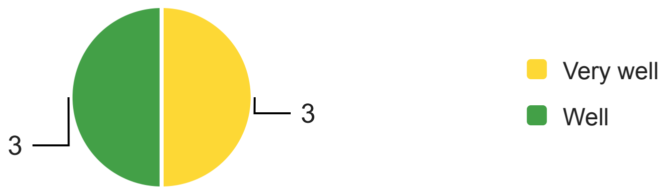

Q38 - How likely are you to try earning new badges?

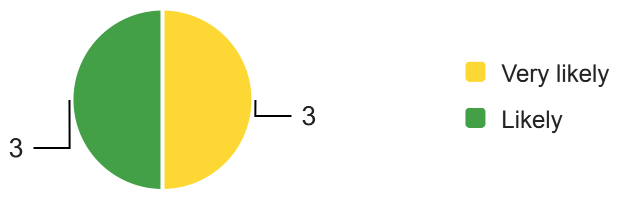

Q39 - How connected do badges feel to challenges or goals?

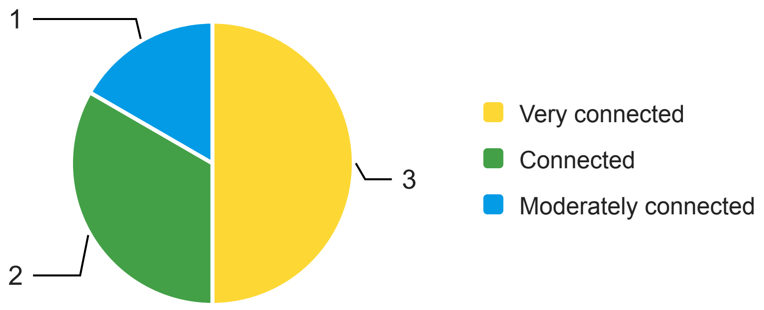

Q41 - How interested are you in joining a group?

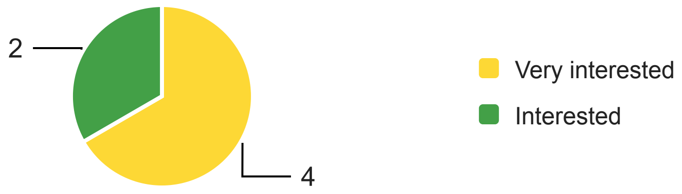

Q42 - How easy is it to find a group that matches your interests?

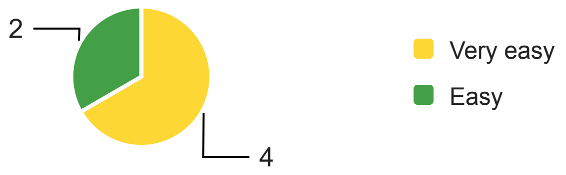

Q43 - How valuable do you think group interaction will be?

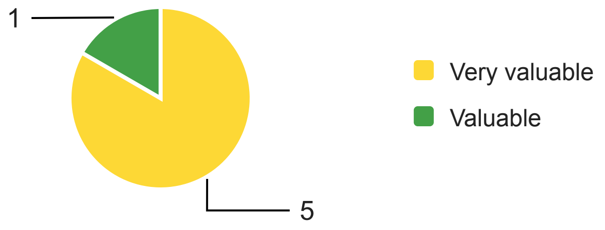

Q44 - How engaging does the groups feature look/feel?

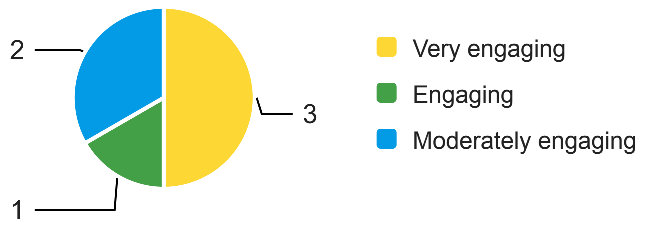

Q45 - How comfortable would you feel joining a group?

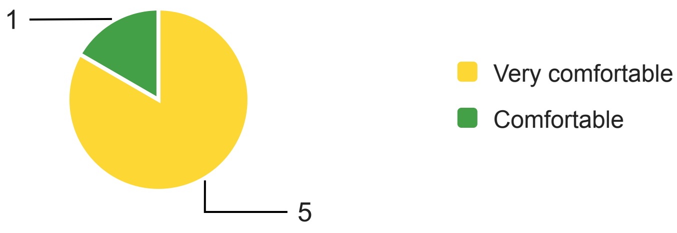

Q46 - Do you have any other thoughts on groups?

Do you have any other thoughts on groups?
No other thoughts.
join one main group during onboarding.<br><br>for finding groups: would be cool to see groups focused on badges or xp or level or other. could be cool to see those other filters.<br><br>i like the groups feature a lot. good overall
Is it just on the app? Is there a message board? It would be cool to allow groups to add a link
What does making your own group look like?
Are group challenges something that all users need to complete? do all users need to stay within the 80% required by the challenge.<br><br>A message board within the group would be cool. kind of like how discord has a channel. people might say "Hey, were working on a group challenge and you haven't done xyz. get your shit done!", or sending a funny.
