# Supplementary material for: Sugar slay: a gamified decision support ecosystem for type 1 diabetes
Source: Front Digit Health. 2026 Jun 17;8:1779790. doi: 10.3389/fdgth.2026.1779790 (PMC13319092; doi:10.3389/fdgth.2026.1779790)
Supplement: Supplementary file 4 [file Datasheet2.pdf]

Northeastern University

Participant Information

Name

Age

When were you diagnosed with T1D?

Onboarding

How clear was the onboarding process?

- Unclear
- Slightly clear
- Moderately clear
- Mostly clear
- Very clear

How confidently do you feel that you would be able to continue using the app without further training?

- Not at all confident
- Slightly confident
- Moderately confident
- Mostly confident
- Extremely confident

How well did the onboarding help you understand key features (e.g., logging, insights, challenges)?

- Not at all
- Slightly
- Moderately
- Mostly
- Completely

How visually engaging did you find the onboarding experience?

- Not at all engaging
- Slightly engaging
- Moderately engaging
- Quite engaging
- Extremely engaging

Do you have any other thoughts on the onboarding experience?

Profile (Gamification Features)

How easy is it to understand your current level/streak?

- Very difficult
- Slightly difficult
- Moderately easy
- Easy
- Very easy

How motivating are badges or levels for you?

- Not at all motivating
- Slightly motivating
- Moderately motivating
- Motivating
- Very motivating

How likely are you to check the profile page to view your progress?

- Not at all likely
- Slightly likely
- Moderately likely

- Likely
- Very likely

Do you have any other thoughts on gamification features?

Glucose Forecast

How easy is it to understand your forecasted glucose trends?

- Very difficult
- Slightly difficult
- Moderately easy
- Easy
- Very easy

How helpful do you find the range presentation?

- Not helpful
- Slightly helpful
- Moderately helpful
- Helpful
- Very helpful

How likely are you to use this feature daily?

- Not at all likely

- Slightly likely
- Moderately likely
- Likely
- Very likely

Do you have any other thoughts on the glucose forecast

Logging Data

How easy is it to log meals?

- Very difficult
- Slightly
- Moderately easy
- Easy
- Very easy

Do you like this form of meal logging?

- Not at all
- Slightly
- Moderately
- Mostly
- Very much

How likely are you to log meals?

- Not at all likely
- Slightly unlikely
- Moderately likely
- Likely
- Very likely

How easy is it to log insulin?

- Very difficult
- Slightly
- Moderately easy
- Easy
- Very easy

How likely are you to log your insulin?

- Not at all likely
- Slightly unlikely
- Moderately likely
- Likely
- Very likely

How easy is it to log your mood?

- Very difficult
- Slightly
- Moderately easy
- Easy
- Very easy

How likely are you to log your mood?

- Not at all likely
- Slightly unlikely
- Moderately likely
- Likely
- Very likely

Do you have any other thoughts on the design of logging data?

Insights & Data

How understandable are the insights provided

- Not at all understandable
- Slightly understandable
- Moderately understandable
- Understandable
- Very understandable

How helpful do the insights feel?

- Not helpful
- Slightly helpful

- Moderately helpful
- Helpful
- Very helpful

How easy is it to navigate between insights?

- Very confusing
- Slightly confusing
- Somewhat easy
- Easy
- Very easy

How visually appealing do you find the data presentation?

- Not at all appealing
- Slightly appealing
- Moderately appealing
- Appealing
- Very appealing

How effective are the visuals in conveying the data?

- Not effective at all
- Slightly effective
- Moderately effective
- Effective
- Very effective

Are there any insights we're missing?

Do you have any other thoughts on the insights feature?

Challenges

How easy is it to understand each challenge?

- Very confusing
- Slightly confusing
- Somewhat clear
- Clear
- Very clear

How motivating are challenges for you?

- Not motivating
- Slightly motivating
- Moderately motivating
- Motivating
- Very motivating

How likely are you to return to continue a challenge?

- Not at all likely
- Slightly unlikely
- Moderately likely
- Likely
- Very likely

How well do challenges fit into your daily routine?

- Not well at all
- Slightly well
- Moderately well
- Well
- Very well

Do you have any other thoughts on challenges?

Badges

How meaningful do badges feel to you?

- Not at all meaningful
- Slightly meaningful
- Moderately meaningful
- Meaningful
- Very meaningful

How well do you understand what badges represent?

- Not well at all
- Slightly well
- Moderately well
- Well
- Very well

How likely are you to try earning new badges?

- Not at all likely
- Unlikely
- Moderately likely
- Likely
- Very likely

How connected do badges feel to challenges or goals?

- Not at all connected
- Slightly connected
- Moderately connected
- Connected
- Very connected

Do you have any other thoughts on badges?

Groups

How interested are you in joining a group?

- Not at all interested
- Slightly interested
- Moderately interested
- Interested
- Very interested

How easy is it to find a group that matches your interests?

- Very difficult
- Slightly difficult
- Moderately easy
- Easy
- Very easy

How valuable do you think group interaction will be?

- Not at all valuable
- Slightly valuable
- Moderately valuable
- Valuable
- Very valuable

How engaging does the groups feature look/feel?

- Not at all engaging
- Slightly engaging
- Moderately engaging
- Engaging
- Very engaging

How comfortable would you feel joining a group?

- Not at all comfortable
- Slightly comfortable
- Moderately comfortable
- Comfortable
- Very comfortable

Do you have any other thoughts on groups?

Branding & Illustrations

How much do you like Bluddy?

- Not at all
- Slightly
- Moderately
- Quite a bit
- Very much

How connected do you feel to Bluddy as a character?

- Not at all connected
- Slightly connected
- Moderately connected
- Connected
- Very connected

How appealing is the illustration style overall?

- Not at all appealing
- Slightly appealing
- Moderately appealing
- Appealing
- Very appealing

How appropriate does the illustration style feel for this app?

- Not appropriate
- Slightly appropriate
- Moderately appropriate
- Appropriate
- Very appropriate

Do you have any other thoughts on Bluddy?

Device Integration / Pairing

How likely would you be to connect a WHOOP band?

- Not likely
- Slightly likely
- Moderately likely
- Likely
- Very likely

How helpful do you think a WHOOP band would be in your experience?

- Not helpful
- Slightly helpful
- Moderately helpful
- Helpful
- Very helpful

How likely would you be to connect a Dexcom?

- Not likely
- Slightly likely
- Moderately likely
- Likely
- Very likely

How important is device integration to your experience?

- Not at all important
- Slightly important
- Moderately important
- Important
- Very important

How likely would you be to use the app WITHOUT integrated devices?

- Not likely
- Slightly likely
- Moderately likely
- Likely
- Very likely

Do you have any other thoughts on device integration?

Powered by Qualtrics
